# Effect of wheat bread with elevated amylose on postprandial glycaemic response: a randomised crossover trial delivered remotely using continuous glucose monitoring

**DOI:** 10.1039/d5fo03147h

**Published:** 2025-11-24

**Authors:** Marina Corrado, George M. Savva, Jennifer H. Ahn-Jarvis, Samson A. Oyeyinka, Cathrina H. Edwards, Brittany A. Hazard

**Affiliations:** a Quadram Institute Bioscience, Norwich Research Park Norwich UK brittany.hazard@quadram.ac.uk cathrina.edwards@quadram.ac.uk; b Department of Nutrition, Food Science and Gastronomy, Faculty of Pharmacy and Food Sciences, Institute of Nutrition and Food Safety (INSA-UB), University of Barcelona Barcelona Spain; c CIBER Physiopathology of Obesity and Nutrition (CIBEROBN), Institute of Health Carlos III Madrid Spain; d School of Agri-Food Technology and Manufacturing, College of Health and Science, University of Lincoln UK

## Abstract

This study measured the effect of white bread made with a *starch branching enzyme II* (*sbeII*) mutant wheat flour with elevated amylose on postprandial glycaemic response compared to an isoglucidic white bread made with a wild-type (WT) control flour. A randomised, double-blind, placebo-controlled two-period crossover trial was conducted to measure glycaemic responses after consuming *sbeII* and WT bread rolls, in duplicates. The study, comprising 26 healthy adult participants (≥18 years of age; BMI ≥ 18 and ≤30 kg m^−2^; HbA1C < 42 mmol mol^−1^, 6.0%), was conducted remotely in the participants’ homes and interstitial glucose concentration was measured by continuous glucose monitors on the upper arm for 10 days. No harms or adverse events were detected; one participant withdrew from the study due to inability to finish the bread roll meal. The maximum rise in glucose within 2 hours did not differ significantly between breads (−0.08 ± 0.12 mMol L^−1^, mean difference ± SE, *p* = 0.514), even though *in vitro* starch digestion was ∼7% lower for the *sbeII* bread than the WT (*p* = 0.006). Effects on satiety and palatability were evaluated using online questionnaires; there was no difference between products in their overall effects on satiety, however more participants preferred the WT bread compared to the *sbeII* bread, which had a slightly harder and less resilient texture when measured instrumentally. Future studies should investigate the dose-dependent effects of foods with increased amylose on glycaemic responses to determine whether higher levels of amylose could yield greater metabolic benefits, while maintaining palatability and consumer acceptance.

## Abstract 2

Postprandial glycaemic response to bread, made with increased-amylase *starch branching enzyme* II wheat mutant, was compared to isoglucidic wild-type bread under real-world conditions using continuous glucose monitors.

## Graphical abstract

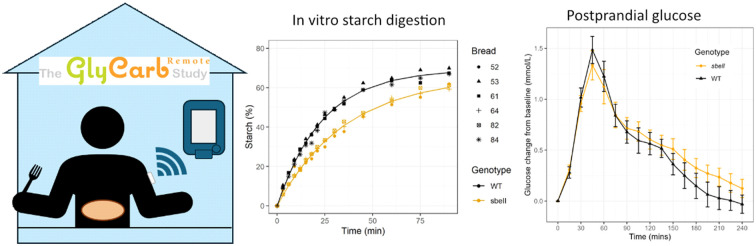

## Introduction

The number of adults living with diabetes has surpassed 800 million, more than quadrupling since 1990, leading to an unsustainable burden on global health services.^1^ A high intake of high-glycaemic starchy foods is a major risk factor for type 2 diabetes, as it contributes to metabolic conditions such as insulin resistance, impaired glucose tolerance, decreased insulin sensitivity, and hyperglycaemia. An important strategy to mitigate dietary risk factors of type 2 diabetes is to produce lower-glycaemic staple foods for consumers.^2^

Wheat (*Triticum aestivum* L. ssp. *aestivum*) is the second most consumed cereal crop (after rice) and provides approximately 18% of the calories consumed worldwide.^3^ Despite the nutritional importance of wheat, staple wheat foods, like bread, are often viewed as unhealthy because they tend to be rapidly digested and typically cause sharp postprandial blood glucose spikes, which, over time, may contribute to impaired glucose tolerance. In contrast, slower-digesting starch leads to a lower glycaemic response.^4^ Since postprandial glucose levels are closely linked to starch digestion in the upper gut, researchers have focused on modifying starch molecular structure in cereal grains, like wheat, to reduce its digestibility in staple foods and enhance their health promoting properties.

The structure of starch can be altered in wheat using TILLING (Targeting Induced Local Lesions IN Genomes) technology to induce mutations in starch biosynthesis genes.^5,6^ For example, wheat with mutations in the *starch branching enzyme II* (*sbeII*) genes results in starch with a higher amylose content leading to greater resistance to amylase digestion compared to normal wheat starch.^7,8^ Such high-amylose starch enables the production of food products, like bread, with lower glycaemic potential without requiring additional processing or supplemental ingredients from other sources.

Prior studies have utilized various wheat mutants differing in amylose contents to examine the glycaemic response to wheat-based foods, resulting in variable findings. For instance, semolina pudding made from a durum wheat [*Triticum turgidum* L. subsp. durum (Desf.) Husn.] *sbeII* mutant (∼35% amylose, compared to ∼23% in the wild-type control) demonstrated reduced starch digestibility *in vitro*; however, its glycaemic index (GI) did not differ significantly from that of the control prepared with wild-type wheat.^9^ Another study on pasta made from a durum wheat *sbeII* mutant showed more promising results; Sissons *et al.* reported that high-amylose pasta (made with ∼58% amylose wheat) reduced *in vitro* starch digestion and had a lower GI compared to pasta made with wild-type wheat.^10^ A few studies have examined glycaemic responses to high-amylose bread products; Belobrajdic *et al.* found that high-amylose bread (made with ∼74.3% amylose flour) reduced postprandial glycaemic and insulinemic responses, but provided less starch and energy per serving than the control bread, making it unclear if the reduction was due to lower glycaemic load or intrinsic starch digestibility changes.^11^ Similarly, Di Rosa *et al.* used high-amylose flour (∼54% amylose) to make a range of wheat foods and showed that high-amylose biscuits and bread, that were not matched for starch content in controls, had significantly lower glycaemic indices than conventional counterparts, but high-amylose Taralli (Italian wheat-based cracker), which had similar starch content as its control, did not have a significantly different glycaemic index.^12^ A study of high-amylose wheat noodles showed a lower glycaemic response, but similarly the served amount of test foods presented different available carbohydrate contents which likely contributed to the reduced postprandial glycemia.^13^ Costabile *et al.* found that bread made with high-amylose/conventional flour blends (85% amylose-rich flour with 15% conventional, and 70% amylose-rich flour with 30% conventional) reduced postprandial glucose response compared to isoglucidic control bread solely made with conventional flour.^14^ While high-amylose wheat mutants show potential for reducing glycaemic response in foods, further research is needed to distinguish the effects of intrinsic changes in starch structure from those of overall available carbohydrates. Additionally, the optimal amylose content and the most effective product types for delivering significant *in vivo* effects to consumers remain unclear.

Furthermore, our prior clinical trial ‘REST’ suggested white bread made with *sbeII* wheat may have low-glycaemic potential, but the full extent of glycaemic impact still required further investigation.^8^ We previously found that bread rolls made with a *sbeII* mutant (∼39% amylose) showed lower starch digestibility *in vitro* (∼20% less) leading to ∼15% lower glycaemic response measured *in vivo*, compared to an isoglucidic WT control (∼26% amylose). However, these estimates were based on data from only eight participants, as the REST study was prematurely terminated due to the COVID-19 pandemic.^8^ In the REST study we also found that blood glucose measurements using capillary bloods from finger-prick samples were comparable to glucose measured in interstitial fluid by continuous glucose monitors (CGMs) on the upper arm.^8^ CGM devices, already widely used by individuals with diabetes, demonstrate strong correlation with traditional methods and have been successfully employed in research.^15,16^ Thus, in this current study we employed CGM technology to conduct a fully remote clinical trial to test whether bread rolls made from *sbeII* wheat (∼44% amylose) reduce postprandial glycaemic responses compared to an isoglucidic wild-type wheat control (∼27% amylose). Unlike a Glycaemic Index study, this postprandial study examined how *sbeII* starch in bread influences the size and shape of the glycaemic response curve, compared with white bread made with WT wheat, to provide insight into the potential health benefits of replacing standard white bread with *sbeII* bread. In addition to glycaemic measures, we evaluated effects of *sbeII* wheat bread on satiety and palatability using online questionnaires. The novelty of this study lies in its implementation within a remote, less controlled setting, which more closely reflects real-world conditions for the introduction and evaluation of novel foods in everyday life. This was the first study being conducted under the GlyCarb Remote study umbrella (ClinicalTrials.gov ID: NCT05317429), a platform designed to test the effect of novel carbohydrate foods on glycaemia using a standardised and robust methodology. As a pilot study within the GlyCarb platform, this work establishes the methodology for future investigations, which will enable direct comparisons across different food structures using a standardized approach.

## Methods

### Bread formulation and process

A *sbeII* mutant bread wheat (*Triticum aestivum* L. ssp. *aestivum*), originally developed by Schönhofen *et al.*,^7^ and a WT control bread wheat (cultivar Lassik) were sown in 2019 in a field trial using a randomised block design at the John Innes Centre's Church Farm field station (Bawburgh, UK). The cultivar Lassik was selected as the control because the *sbeII* mutant was generated in this genetic background. Wheat grains were bulked and milled with ∼16.4% humidity at ADM Milling Limited (Bristol, UK). Grains were de-branned and milled on a Bühler mill with a feeding rate of 100 g min^−1^.

The *sbeII* and WT control wheat bread rolls were produced at the Quadram Institute (NHS QI Clinical Research Facility, Norwich, UK). The formulation of each bread was adjusted based on the flour's starch content so that both provided the same total starch (∼72 g) per served portion. Briefly, flour (54.7% and 56.7% for *sbeII* and WT control, respectively), yeast (1.6%), sugar (2%), salt (1%), Trex shortening (1.8%), and water (38.9% and 36.9% for *sbeII* and WT control, respectively) were mixed at low speed for 3 min and kneaded for 5 min at increasing speed, in a heavy-duty planetary mixer with a hook attachment (model 5KSM7591XBSM, Kitchen Aid, Antwerp, BE). Dough was fermented for 2 h at 21 °C then portioned into rolls and proofed for 15 min at 38 °C with 100% relative humidity, then baked using a combination oven (Rational Self Cooking Centre, Luton, UK) for 15 min at 185 °C (40% humidity for 10 min and 10% humidity for the last 5 min). Baked rolls reached ∼95 °C core temperature. After 2 h cooling at 21 °C, breads were packed in resealable, opaque polyethylene bags (3 mm thickness) and stored at −20 °C. Breads were produced in four batches of 12 rolls (48 rolls per type of flour), under identical conditions and paired by their baking position in the oven. Rolls were thawed for 14 h to 16 h at room temperature and either served to study participants as part of their breakfast meal or used for *in vitro* analyses. Breads were consumed within eight months of manufacture.

### Bread characteristics and *in vitro* amylolysis

#### Nutrient composition and microbiological safety

Two pairs of bread rolls (*sbeII* and WT control) were randomly selected for proximate nutritional analysis and microbiological safety testing, performed by UKAS accredited testing at ALS Laboratories Ltd, Chatteris, Cambridgeshire, UK, (method references are listed in SI Tables 1 and 2). Bread rolls were deemed safe for human consumption; details can be found in SI Table 1.

#### Starch characteristics

Total starch content of flour was measured using ‘Total starch kit’ (KTSTA-100A DMSO format, AOAC 996.11, Megazyme International, Wicklow, Ireland), *n* = 4, as described in Corrado *et al.* 2023.^17^ Amylose determination was carried out using an iodometric method on starch isolated from *sbeII* and WT white wheat grains as described by Corrado *et al.* 2020,^9^ and reported in Corrado *et al.* 2023.^17^

The proportion of digestible and resistant starch in flour and bread was determined using the reagents from ‘Total starch kit’ and ‘Resistant starch kit’ K-RSTAR (AOAC 2002.02, Megazyme International, Wicklow, Ireland), using a small-scale version of the method, as described by Edwards *et al.* 2015.^18^ Four technical replicates were used in the analysis of starch characteristics of the flour. Four technical replicates were taken from two breads for each genotype (*sbeII* and WT). Moisture was determined using the AACC (44-15A) air oven method, one stage procedure.

### Bread texture

Texture was measured instrumentally using a ‘two-bite test’ on a TA-XT2 Texture Analyser (Stable Micro Systems, Godalming, UK), equipped with a 5 kg load cell using a modified AACC method 74-09, and a 50 mm diameter compression plate (P50). Uniaxial compression with crosshead speed of 100 mm min^−1^ was applied to 25 × 25 × 25 mm crumb samples to mimic mastication, with crumb hardness corresponding to the force (*N*) required for 40% compression. Exponent (version 6.0, Stable Micro Systems, Godalming, UK) software for texture profile analysis was used to assess the following texture parameters: *hardness*, *springiness*, *cohesiveness*, *chewiness* and *resilience*. A separate linear model was estimated for each texture parameter following log-transformation. The texture parameter *springiness* was highly variable between replicates and therefore difficult to model. No statistical model was fitted for this outcome.

#### *In vitro* starch susceptibility to amylase

Starch susceptibility to amylase digestion (amylolysis) was determined using an established amylolysis assay method based on enzyme-kinetic principles, previously reported to correlate well with Glycaemic Index of foods.^19^ Analysis was performed on three bread rolls per type, using three technical replicates from each roll. Sieved bread crumb was weighed in a tube to achieve 27 mg of starch in 5 mL of Phosphate-Buffered Saline (PBS). Samples were incubated at 37 °C with end-over mixing and 2 U mL^−1^ of porcine pancreatic α-amylase per incubation mix were added to start the assay, after taking a baseline measurement of endogenous maltose (*Y*_0_). Samples of incubation mix (100 μl) were taken at 0 (before adding the enzyme), 3, 6, 9, 12, 15, 18, 21, 25, 30, 35, 45, 60, 75, 90 min of incubation and added to tubes containing 100 μL of Na_2_CO_3_ to stop the reaction. Reducing sugars obtained from hydrolysis were measured using a ‘PAHBAH’ (*p*-hydroxybenzoic acid hydrazide) colorimetric method, as described by Edwards *et al.* 2019.^19^ Samples were centrifuged at 15 000*g* for 5 min; the supernatant was diluted 1 : 10 with deionised water to 100 μL and incubated in a boiling water bath for 5 min with 1 mL of PAHBAH reagent. Samples and standard solutions (0–1 mM maltose in water) were incubated with PAHBAH reagent. Absorbance was read in a microplate reader (VersaMax Microplate Reader, Molecular Devices, LLC., CA, USA) at 405 nm and maltose equivalent concentration in samples were calculated using the maltose standard curve.

Amylolysis curves were baseline corrected by subtracting the value at *t* = 0 min (*Y*_0_) from each replicate. Starch digestibility was expressed as the percentage of starch digested *C*_90_ representing extent of digestion after 90 min of incubation with α-amylase. Experimental data points were fitted to a first order equation (eqn (1)) previously described^19^ where *C*_*t*_ is the extent of starch digested a *t* min, *C*_∞_ is the predicted extent of starch digested at the reaction end-point, and *k* is the digestibility rate constant.

1 *C*_*t*_ = *C*_∞_(1 − *e*^−*kt*^)

Non-linear regression was used to obtain *k* and *C*_∞_. The effect of genotype (*sbeII* and WT) on starch digestibility parameters (*Y*_0_, *C*_90_, iAUC, *k* and *C*_∞_) were estimated from the model and compared between groups.

#### Sample preparation

Three pairs of each bread type were randomly selected for *in vitro* analysis of starch characteristics. From each roll, a minimum of three technical replicates were sampled for analysis, as specified below. Rolls were left to thaw in their packaging at room temperature (∼21 °C) for 16 h; after removing the crust they were sliced into four cubes per roll for texture analysis. Following texture analysis, the cubes were ground using a food processer (Kenwood CH 180 Mini chopper) for 50 seconds and sieved, either to pass through a 1 mm screen in preparation for moisture determination and *in vitro* amylolysis assay, or to pass through a 500 µm screen in preparation for measurement of digestible and resistant starch. These were weighed into dry-tared aluminium pan in triplicates for moisture determination, into 2 mL safe lock tubes (STARLAB, UK) in quadruplicates (technical replicates) for digestible and resistant starch determination, and into 15 mL Corning centrifuge tubes (Merck KGaA, Darmstadt, DE) in triplicates for amylolysis analysis.

### Acute postprandial human intervention study

#### Study design

The study was conducted according to the guidelines of the Declaration of Helsinki, and all procedures involving human subjects were approved by the Cambridge South Research Ethics Committee (REC reference 21/EE/0245); the study protocol was registered as QIB07/2020. The trial was registered on ClinicalTrials.gov (ID: NCT05317429) on 16^th^ February 2022.

A double-blind randomised crossover study was conducted in which healthy human participants (*n* = 26) were instructed to consume bread meals made from WT (control) or *sbeII* wheat while fitted with CGMs for remote measurement of glycaemic responses.

The study was conducted remotely during February to October 2022 with test meal consumption, glucose monitoring, satiety and palatability questionnaires and other study procedures performed by participants in their homes. Interactions between the study team and participants were through virtual appointments, participants gave verbal informed consent *via* recorded video call and written informed consent *via* post, and study equipment and meals were provided through the post.

Participants consumed each bread meal type (WT control and *sbeII*) twice on separate breakfast occasions after an overnight fast, with a washout of one day between meals. The study team enrolled participants and the meal order was assigned using block randomisation to ensure a balanced design and avoiding the potential for an order effect. The allocation sequence was generated in R; four sequences (with treatment order ABBA, BAAB, AABB, BBAA) were allocated in three blocks of 8, with the final two participants assigned a sequence at random. All study meals were consumed within a 15-day CGM monitoring period.

Participants were guided to self-apply CGMs two days prior to scheduled test meal consumption. On the evening prior to the scheduled test meal consumption, participants were requested to avoid alcohol, strenuous exercise and/or high caloric meals, to drink plenty of water and fast overnight (taking only water). Participants were instructed to take meals out of the freezer 12–14 h before consumption. The following morning, fasted participants would prepare the assigned test meal breakfast, which consisted of one bread roll providing ∼72 g of total starch (total of both digestible and resistant starch, Table 1), either from *sbeII* or WT control wheat flour, 10 g of vegetable spread (Flora) and approximately 250 mL of water to drink. The nutritional composition of the vegetable spread is reported in SI Table 3. The breads had a similar appearance and were labelled with a code so that participants did not know which bread type they were consuming. The time at which meal consumption commenced was recorded by participants. Participants completed online questionnaires (Microsoft Forms, questions listed in SI Table 4) on satiety (before and after each meal), and palatability (after each meal), while the fitted continuous glucose monitors (Abbott FreeStyle Libre Flash Glucose Monitoring System) collected interstitial glucose measurements. For the 4 h after eating the test meal, the participants were requested to refrain from eating or drinking anything other than water and limit any physical exercise.

Bread nutritional composition and starch characteristics per serving (fresh weight basis)

**Table:** 

Nutrients per serving	WT control bread meal	*sbeII* bread meal
Energy^a^ (kcal)	369.4	375.6
Energy (kJ)	1562.2	1587.0
Protein^b^ (g)	16.0	18.8
Total fat^c^ (g)	4.3	4.7
Saturated fat (g)	1.3	1.4
Poly-unsaturated fat (g)	1.2	1.4
Ash	2.2	2.8
Available Carbohydrates^d^ (g)	64.6	61.5
Total sugars^e^ (g)	0.8	0.9
Fibre AOAC^f^ (g)	4.0	6.2

**Table:** 

Bread characteristics	WT bread	*sbeII* bread
Bread roll serving (g)	159.9 ± 0.3	172.3 ± 0.2
Moisture (%)	45.7 ± 0.3	47.7 ± 0.2
Total starch (g)	72.2 ± 0.18	72.3 ± 0.08
Digestible starch (%)	95.3	90.4
Resistant starch (%)	4.7	9.6

**Table:** 

*In vitro* amylolysis	WT bread	*sbeII* bread
*Y*_0_ (%)	1.7 [1.3; 2.2]	2.9 [2.5; 3.4]
*C*_90_ (%)	68.1 [65.8; 70.4]	60.8 [58.5; 63.2]
*C*_∞_ (%)	69.2 [66.2; 72.4]	65.5 [62.6; 68.5]
*k* (min^−1^)	0.042 [0.038; 0.045]	0.028 [0.025; 0.030]
iAUC (% min^−1^)	4616 [4442; 4790]	3733 [3559; 3907]

a Calculated using standard energy conversion factors (EC 2008/100 and 90/496).

b Total nitrogen for a given sample is measured by combustion, nitrogen-to-protein conversion factor = 6.25

c Determined by NMR.

d Determined by ion exchange chromatography, calculated by difference.

e Determined by ion exchange chromatography.

f AOAC method 985.29 (‘Prosky method’), which includes RS type 3 (retrograded starch).

#### Study participants and sample size estimations

Previous published research^15^ showed that to detect a relative difference of 15% in incremental glucose concentration (iCmax) between carbohydrate-based meals, 20 participants consuming each meal twice were required for a power of 80% at *p* < 0.05. This was based on a SD of 0.7 for treatment difference between individuals from a two-period study with a mean average 2.5 iCmax in the control group. To estimate the between-participant variation in treatment difference for a four-period study we assumed that 30% of the observed between-participant variance was attributable to genuine between-participant treatment effect differences and 70% to day-to-day differences in iCmax and its measurement. To account for potential dropouts, we aimed to randomise a minimum of 25 participants.

Participants were recruited from within approximately a 40-mile radius of the Quadram Institute through poster and e-mail advertisements circulated across the Norwich Research Park. These materials invited individuals interested in receiving further information about the study to contact the named researchers and complete an expression of interest form. Potential participants were then asked to complete an online pre-screening questionnaire. Those who met the eligibility criteria based on their responses were invited to attend a virtual consent and screening visit. Participants meeting the following criteria were deemed eligible to take part: ≥18 years of age who have access to a smartphone and/or tablet or computer and were willing to use this for the study, BMI ≥ 18 and ≤30 kg m^−2^, and HbA1C < 42 mmol mol^−1^ (6.0%). Participants were excluded if they were smokers, allergic or intolerant to the study foods or to adhesives, were alcohol or substance abusers or had insulin-dependent or non-insulin dependent diabetes, gastrointestinal disorders, anaemia, cardiovascular disease, active cancer or taking medications that could have affected the study outcome. Women who were pregnant, lactating, or had given birth during the last 12 months were excluded, as well as individuals participating in other intervention studies, and those related or living with any members of the study team or unable to provide written informed consent. All participants gave their full informed written consent prior to taking part.

### Statistical methods

#### Acute postprandial intervention study

The predetermined primary outcome measure was the maximum rise in interstitial fluid glucose in the two-hour period immediately following the meal. The baseline (fasted glucose value) was taken as the reading that was the latest value that was still at least fifteen minutes before the participant stated that they had started the meal. The incremental area under the glucose curve between 0 and 2 h was used as a secondary outcome measure with any areas below the baseline value discarded. Participants reported varying meal start times, while glucose measurements were taken at fixed intervals, preventing direct alignment with standard postprandial time points. Thus, values at 0 minutes and at 15-minute intervals up to four hours post-meal were interpolated for each glucose curve from the observed values using the approx function in R.

For each outcome the difference between bread types was estimated using a linear mixed model, with the treatment effect and meal order as fixed effects, and a random participant specific intercept and random participant specific treatment effect (random slope model). Mixed models were estimated using lmerTest for R.^20,21^ Missing glucose traces were considered to be missing at random as they arose from technical issues.

Satiety and sensory outcomes were assessed using questions with ordinal responses (SI Table 3). For data analysis, Spearman's rho was used to estimate correlations between responses to different questions within each construct at each time point. For each outcome a one factor model confirmatory factor analysis was used to estimate single underlying latent variables for each construct (satiety at each time point and overall liking). Wilcoxon paired tests were used to test for differences between *sbeII* and WT bread meals on overall satiety and for individual satiety questions immediately following the meal and during the 4 hours after. For sensory variables, sign tests were used to compare the proportion of individuals who preferred each bread (based on the average overall liking score) between breads, and a random slope model similar to that used for glucose measures was used to compare the latent ‘liking’ score generated from the confirmatory factor analysis.

## Results

### Flour characteristics

As reported in Corrado *et al.* 2024 ^22^ the total starch content of the *sbeII* flour (65.5% ± 1.5%) was slightly lower than the WT control flour (69.4% ± 1.0%) (mean ± SEM, *n* = 4) and the apparent amylose proportion measured by iodine-binding method on starch isolated from flour was greater for *sbeII* starch (43.5 ± 0.4% of total starch, mean ± SEM, *n* = 3) than the WT control (27.2 ± 2.0% of total starch, mean ± SEM, *n* = 3). Flour proximate analysis was previously reported^22^ and is included in SI Table 2 for ease of access.

### Bread characteristics

**Fig. 1:**
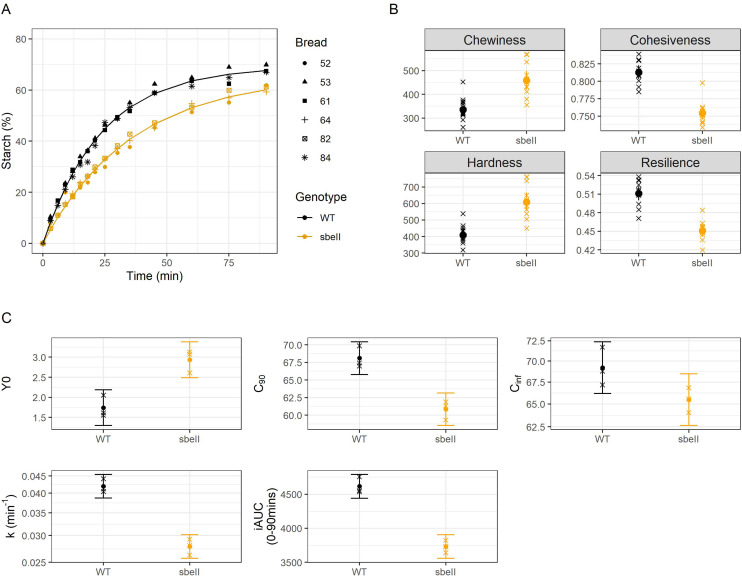
A. Starch susceptibility to amylolysis curves, *sbeII* (yellow) and WT control (black) bread samples, experimental data points represent independently treated samples, *n* = 3 loaves. Experimental data (replicate datapoints) are shown by fitting a first-order equation based on the estimates of *k* and *C*_∞_ values (*n* = 3 independent samples) obtained from a non-linear regression model. B. Texture parameters measured instrumentally by Texture Analyser. C. Grouped means of parameters obtained from digestibility curves, *Y*_0_, *C*_90_, *C*_∞_, *k*, iAUC, error bars represent the 95% CI (*n* = 3).

The average serving size of *sbeII* breads was 172.31 g ± 0.26 g (fresh weight) and the average size of WT control breads was 159.90 g ± 0.26 g (fresh weight), (mean ± SEMs, *n* = 84 rolls per type). Measured nutrient composition, indicators of *in vitro* amylolysis and starch characteristics of bread rolls are reported in Table 1. The total starch contents of the final test breads were 72.2 ± 0.18 g for the WT control and 72.3 ± 0.08 g for the *sbeII* bread – these values were calculated using the measured starch content of the flour, which is considered a more reliable basis than the ‘by difference’ calculations from proximate analyses. *In vitro* digestion of *sbeII* bread resulted in a lower proportion of starch digested after 90 min of incubation with α-amylase (*C*_90_ = 60.8%%) compared to the WT control (*C*_90_ = 68.1%, *p* = 0.006). The rate of digestion was slower for *sbeII* starch (*k* mean difference between groups = −0.41 min^−1^), resulting in a lower iAUC (iAUC mean difference between groups = −883% min^−1^, *p* < 0.001). Amylolysis curves and digestibility parameters are shown in Fig. 1A and C. Fig. 1B shows texture parameters measured instrumentally. There was a small but significant difference in crumb hardness (*p* < 0.001) and resilience (*p* < 0.001) between bread types.

### Acute postprandial human intervention study participants

**Fig. 2:**
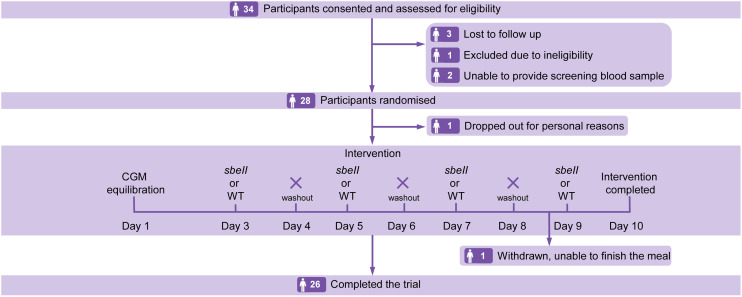
Study flow diagram based on the Consolidated Standards of Reporting Trials (CONSORT).

A total of 28 participants were randomised to the study. Of these, one participant dropped out for personal reasons. One participant withdrew due to inability to finish the meal portion; data from this participant was excluded in the analysis. The remaining 26 participants completed the study (Fig. 2).

Glucose responses from some meals (*n* = 4) were excluded because of technical problems with the CGM. Hence the final analysis dataset for glucose response outcomes included 26 participants, with datasets from 22 participants including four meals (two *sbeII* and two WT per participant) and four participants including data from three meals (including at least one of each product per participant). Hence, glucose data from 100 meals in total, with 50 from each treatment, were included in the final analysis.

Participant characteristics are reported in Table 2. Participants were aged between 18 and 64 years of age. Of the 26 participants that completed the study, 46% were between 25–34 years old, 19% were between 45–54 years old, 15% were 35–44 and 55–64 years old, and 4% were 18–24 years old. Participants had the study meal between 07:00 and 10:00.

**Table 2:** Baseline characteristics of participants that completed the study

Characteristics	Mean	SD	Min	Max
Height (cm)	172.4	11.7	157	203
Weight (kg)	68.6	11.4	51.6	94.3
BMI (kg m^−2^)	23.0	2.8	18.4	29.5
Body Fat (%)	27.6	9.8	11.5	44.3
Body Muscle (%)	81.0	245.1	23.9	1306.0
Resting metabolism (kcal)	1481.0	188.0	1187.0	1890.0
Visceral Fat (score)	5.0	2.4	1.0	12.0
HbA1c (mmol mol^−1^)	32	3.9	25	40

No harm or adverse events were detected. One participant withdrew from the study due to inability to finish the bread roll meal.

### Glycaemic responses

**Fig. 3:**
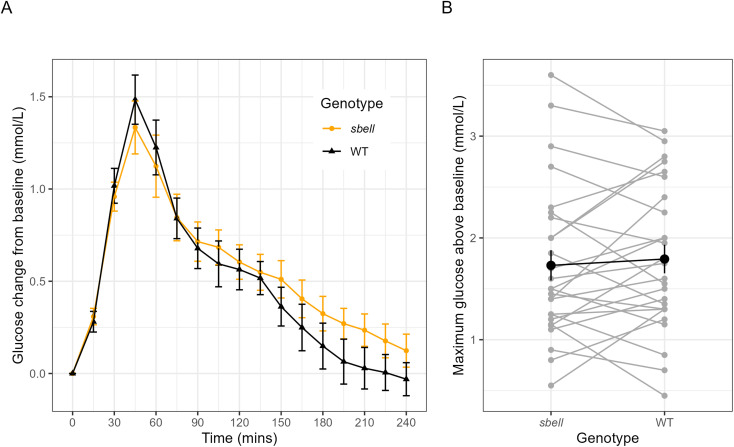
A. Average glucose values by *sbeII* and WT bread treatment, corrected for baseline (latest glucose reading taken at least 15 minutes before the stated mealtime). Error bars show ± 1 standard error. B. Paired glycaemic responses (gray), showing the average maximum increase in glucose level per participant for each bread type, paired by participant. The average across participants is highlighted in black.

The average glycaemic response between the *sbeII* and WT bread meals is compared in Fig. 3A. The curves do not significantly differ at any time point, although the response to the *sbeII* bread has a slightly lower peak, while in the later part of the curve the response to the *sbeII* bread appears slightly higher (from 2 to 4 h).

Table 3 shows the mean of each outcome measure for *sbeII* and WT bread treatments and estimates for the mean difference in absolute terms and as a proportion of the average response to WT bread. The primary outcome measure was the maximum glucose change from baseline (fasted glucose value) over two hours following the meal (iMax120), with the baseline (fasted glucose) considered as the last glucose reading taken at least 15 minutes before each meal. This did not vary significantly between groups (mean (se) difference: −0.08 (0.12) mMol L^−1^, as a relative change: −4.57% (6.96), *p* = 0.514). Fig. 3B shows average iMax120 for each participant following each bread, paired by participant, as well as the average across participants. Likewise, there was no statistically significant difference in AUC at either 120 minutes or 240 minutes.

**Table 3:** Measures of glycaemic response by meal up to four hours after meal

Measure	*sbeII* (mmol L^−1^), Mean (sd)	WT (mmol L^−1^), Mean (sd)	Treatment effect (se) (mmol L^−1^)	Effect as % of WT (se)	*P*-value
Fasted glucose	4.57 (0.46)	4.66 (0.45)	−0.10 (0.06)	−2.20 (1.35)	0.115
iMax120	1.73 (0.76)	1.79 (0.72)	−0.08 (0.12)	−4.57 (6.96)	0.514
iMax240	1.76 (0.75)	1.82 (0.69)	−0.09 (0.12)	−4.68 (6.84)	0.497
iAUC120	99.24 (47.39)	105.07 (50.34)	−7.56 (9.15)	−7.20 (8.71)	0.416
iAUC240	152.76 (69.98)	154.46 (67.00)	−4.68 (14.45)	−3.03 (9.35)	0.749

CGM data showed marked variability in glucose concentrations leading up to the self-reported breakfast times. Despite overnight fasting, many participants showed a rise in glucose before reporting meal initiation. This pre-prandial increase varied across individuals and days, contributing to baseline variability. SI Fig. 1 shows glucose traces illustrating these highly variable pre-breakfast patterns.

There was no evidence that the time to maximum glucose (tMax) value varied between breads (median = 53.25 min for both meals). Fasted (baseline) glucose levels did not significantly differ between breads, although were on average slightly lower prior to the sbell bread (mean = 4.57 mmol L^−1^, sd = 0.46) compared to the wild type (mean = 4.66, sd = 0.45). There was an order effect evident for some outcomes, glucose traces appeared to increase over the study period, with glucose level an average 0.36 mmol L^−1^ higher (se = 0.06, *p* < 0.01) during the final test period compared to the first. This did not affect the estimation of the difference between breads, since the breads were balanced over time points by the study design, outcomes were typically corrected for a pre-meal level and regression models adjusted for meal order.

Variance components from the random slope models are shown in SI Table 5. There was little evidence for a varying treatment effect across participants for the peak glucose, but for iAUC there was evidence for a substantial difference in the effect of different breads across participants, suggesting that there may have been some effect for some participants, despite the mean effect being difficult to detect in the present study.

#### Satiety responses

**Fig. 4:**
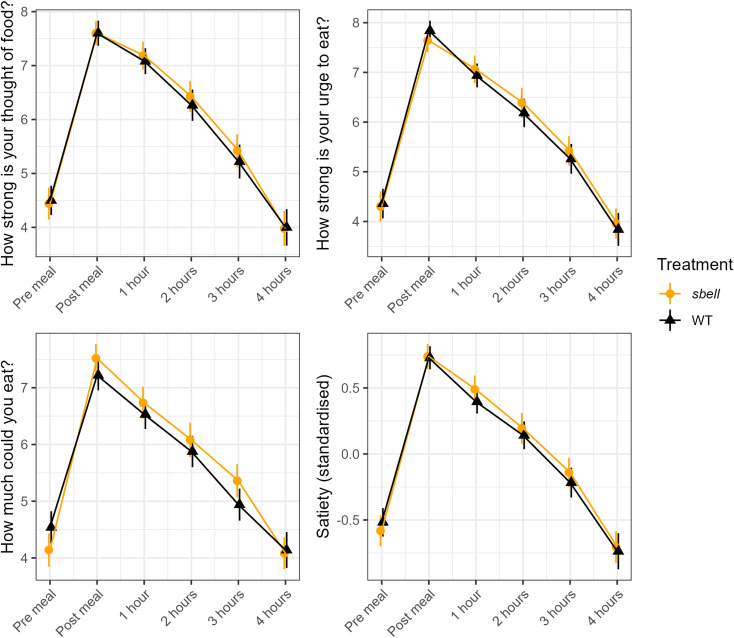
Postprandial satiety response to sbeII and WT breads. Points represent the mean average and standard error of responses for each treatment. Higher scores represent less desire to eat. Each question was recorded on a nine-point scale. The final plot represents a standardised composite score of the three previous questions.

Fig. 4 shows the average satiety scores across bread groups for each of the three questions used for its assessment, and for a standardised variable combining each of the three measures. For both *sbeII* and WT breads, average satiety returned to the pre-meal state between three and four hours. Responses to the three questions were extremely highly correlated at each time point (Spearman's rho between each pair > 0.8), and so a single composite variable was created using a confirmatory factor analysis. There was no evidence for any difference between products in overall satiety either immediately following each meal (*p* = 0.885, Wilcoxon paired test comparing the standardised satiety score) or in the four hours afterwards (*p* = 0.369, 0.614, 0.479, 0.799 for Wilcoxon tests at each time point).

#### Sensory measures

**Fig. 5:**
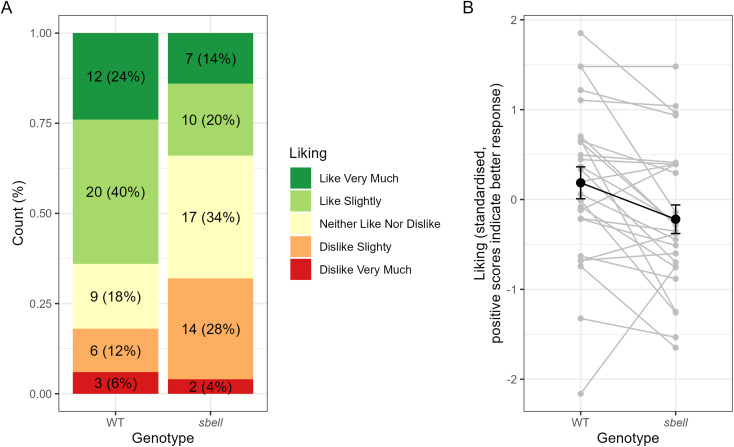
A. Distribution of participants overall liking of *sbeII* and WT bread. B. Standardized liking scores (gray), paired by participant. The average across participants is highlighted in black.

Fig. 5A shows the distribution of participants overall liking of each bread. Significantly more participants reported preferring the WT bread (*n* = 17) compared to the sbeII bread (*n* = 4), with five participants reporting the same overall score for both (*p*-value for binomial test = 0.007).

Scores for other sensory questions, that is flavour, sweetness, bitterness and aroma and how often the participant might eat the bread were highly correlated with overall liking and showed a similar pattern of preference toward the WT bread (SI Fig. 2). A confirmatory factor analysis supported a single factor solution, with very high concordance between each sensory question. Hence a single standardised latent factor representing ‘liking’ was generated using data from all sensory questions, this showed a significantly lower score for the *sbeII* bread compared the WT bread (random slope model, *β* = −0.412, se = 0.14, *p* = 0.008) (Fig. 5B).

## Discussion

In this study, we investigated the glycaemic-lowering potential of white bread made with *sbeII* wheat flour with elevated amylose (43.5% amylose starch) compared to an isoglucidic control made with WT flour (27.2% amylose starch). Despite lower starch digestibility of the *sbeII* bread observed *in vitro*, we did not observe a significant difference in postprandial glycaemic response as measured by maximum glucose concentration within 2 hours after the meal (our primary outcome measure). This is in line with a study by Behall and Hallfrisch which aimed to determine the amylose level required to reduce glucose or insulin levels using breads made with high-amylose corn starch blends.^23^ They found that peak glucose response was lowest after consuming breads containing 50–70% amylose starch and saw no physiological differences for 30% or 40% amylose formulations and concluded that an amylose content above 50% in a single meal is necessary to significantly lower plasma glucose.^23^ However, high amylose wheat and corn starch, despite similar apparent amylose contents, can differ in the proportion of long amylopectin chains which could influence starch digestibility.^24^ In our study, amylose content was measured using the iodine-binding assay, which may also capture contributions from long amylopectin chains, an important consideration in light of other findings on *sbeII* starch showing increased long amylopectin chains.^17,25^

A notable observation in our study was the high variability in glucose levels during the pre-prandial period. While some fluctuation is expected due to natural physiological processes, we observed a pre-prandial rise in glucose likely reflecting anticipatory responses. How this early increase should be accounted for when estimating postprandial responses requires further consideration. It is worth noting that previous studies investigating glycaemic response to high-amylose foods,^10–14^ have primarily relied on traditional capillary and venous blood sampling methods, which limit the number of samples taken over 2–3 hours, and typically report glycaemic index or AUC as the primary outcome measure for glycaemic response. In contrast our study utilized CGM technology allowing us to measure glucose continuously over 10 days resulting in a much greater amount of data. CGMs measure glucose in interstitial fluid, which does not follow the same kinetics as capillary or venous blood traditionally used in glucose studies. Furthermore, in the context of a remote study relying on participant-reported mealtimes, there is also inherent ambiguity in the exact timing of meal initiation. A common challenge in remote studies is the reduced control over study procedures and inconsistent timing of meal commencement can impact the accuracy of time-series data. To improve procedural consistency, future studies should consider incorporating supervised meal initiation.

Compared to our prior study (REST)^8^ on *sbeII* bread we see a consistent trend in slightly lower glycaemic response despite no significant results. Other studies have reported glycaemic lowing potential of high amylose bread, but it is important to consider that the test foods studies generally provided different amounts of available carbohydrate which could influence glycaemic outcomes.^11,12^ Di Rosa *et al.* examined three different food products made from high-amylose wheat.^12^ Test foods that were not matched for starch content, such as biscuits and bread, showed a significantly lower glycaemic response.^12^ However, high amylose taralli crackers, which had a similar starch content to the control taralli, showed no difference in glycemic response.^12^ Vetrani *et al.* also found that consuming amylose-rich rusks significantly reduced glycaemic response compared to conventional rusks in standard meals matched for available carbohydrate in a study of overweight individuals.^26^ While our study matched breads for starch content, in practice, consumer products are often formulated and consumed on an equal-weight basis, making it important to consider this approach as well. Thus, in future research and applications of high amylose wheat it will be important for mechanistic understanding to distinguish effects of grain composition (amount of total available carbohydrate in wheat flour) *versus* intrinsic changes in starch structure.

It is also important to consider that food formulation, as well as cooking and storage conditions, affect the extent of starch gelatinisation and retrogradation in foods, which in turn could influence starch digestibility and glycaemic response. We previously reported that wheat bread made from *sbeII* mutant flour and stored under different conditions (room temperature, refrigeration, or freezing), had significantly higher starch digestibility compared to freshly baked bread.^17^ For practical reasons, participants in the present study received breads in frozen form and thawed them overnight for their breakfast meal. It is worth noting that freezing can alter starch digestibility compared with freshly baked bread. In line with our prior studies, we confirmed that *sbeII* wheat flour leads to lower starch digestibility in white wheat bread measured *in vitro*. However, the difference in digestibility between *sbeII* and WT bread was smaller in this study (difference in *C*_90_: 7.3% *vs.* 13.03% in the REST study^8^), despite similar differences in resistant starch (difference in resistant starch was 4.9% in GlyCarb and 4.7% in REST). The difference in amylose content was slightly greater in this study (16.3% in GlyCarb and 13.3% in REST), which is likely due to environmental factors since grain was produced in different field seasons. This is also interesting as it shows that flours with higher amylose content than conventional wheat may still result in similar resistant starch content and that this does not necessarily lead to lower starch digestibility. This has implications for wheat breeding and food production as high amylose wheats will need to reach a minimum threshold to influence glycaemic outcomes for consumers.

Furthermore, when comparing studies of foods made with high-amylose flours, it should be recognised that flours with the same amylose content can yield foods with markedly different starch digestibility depending on processing. For instance, Sissons *et al.*^10^ reported that pasta made from a *ss2a* mutant with an amylose content equivalent to that in our current study (43.5%) contained ∼2% resistant starch in dry pasta, compared to ∼7% in our frozen and thawed *sbeII* bread rolls; they also reported no significant change in glycaemic index of the cooked *ssIIa* pasta compared to the wildtype control. We also reported that semolina made from a *sbeII* durum wheat mutant with 33% amylose consistently had more resistant starch than the wild type, however, when measured in raw semolina, after gelatinisation (a porridge), and following retrogradation (a pudding), the amount of resistant starch varied depending on the hydrothermal processing applied.^9^

There was no noticeable difference in overall satiety between *sbeII* and WT bread, either immediately after each meal or within the following four hours. This aligns with our previous study, REST, which used VAS (Visual Analogue Scale) questionnaires in a clinical setting and found no differences in the participants’ reported ‘desire to eat’ and ‘hunger’ after consuming *sbeII* and WT breads.^8^ However, REST did provide some evidence of increased ‘fullness’ up to 30 minutes after eating *sbeII* bread. Similarly, the Belobrajdic *et al.* study on high-amylose breads found no differences in subjective satiety during the postprandial period between low- and high-amylose breads.^11^ Gondalia *et al.* also reported no differences in satiety indicators following a two- and four-week feeding study comparing high- and low-amylose breads.^27^ Other studies have suggested increased satiety after consuming wheat products with higher amylose and resistant starch content. For example, Vetrani *et al.* found the desire to eat significantly lower during four hours after a meal incorporating high amylose rusks, but no difference was observed for other subjective appetite measures.^26^ Hughes *et al.* also found no significant effects of bread rolls with higher RS on self-reported appetite measures (VAS), yet they observed changes in satiety-related hormones GLP-1 and PYY linked to gastric emptying, which influence satiety perception.^28^ Similarly, Robertson and Al-Mana demonstrated that RS significantly reduced food intake during an *ad libitum* meal in overweight/obese men compared to a placebo, although it did not significantly affect subjective satiety or hunger.^29^ Conversely, Luhovyy^30^ found no differences in subjective satiety or food intake after consuming cookies made with high-amylose maize flour. Overall, while many studies have not reported changes in subjective satiety, findings on food intake effects remain inconsistent. Further research with larger sample sizes is needed to better understand the impact of increasing amylose in wheat bread and other foods on satiety.

Few studies have examined consumer acceptability products made with high amylose wheat flour. Blasi *et al.* found that high-amylose biscuits are accepted despite some sensory differences.^31^ Results of our sensory analysis showed that more participants preferred the WT control bread compared to the increased amylose *sbeII* bread. This is likely due to changes in the texture of *sbeII* bread. Previous studies, including our own, have consistently demonstrated the impact of *sbeII* mutations on textural characteristics, highlighting the need for formulation optimization to enhance consumer acceptance and end-use quality.^9,17,22,32^ Additionally, various *sbeII* mutants have been reported, leading to a range of amylose levels that may influence the extent of their effect on bread quality.

Our findings provide strong evidence of participant-specific responses to bread meals, which is unsurprising in a remote study like this one. Even in more tightly controlled trials, substantial variations in postprandial responses among individuals are largely influenced by modifiable factors, such as habitual diet, sleep, and exercise, as well as genetic and metabolic (microbiota) factors. This was observed in the large-scale clinical trial PREDICT 1, which aimed to quantify and predict individual differences in postprandial triglyceride, glucose, and insulin responses to standardized meals.^33^ These findings underscore a crucial consideration in the context of novel carbohydrate food development: is the product truly effective at lowering glycaemic response, or is the normoglycaemic population too heterogeneous to treat as a single group? More research is certainly required on this topic.

In conclusion, our study found that bread rolls made from *sbeII* wheat with increased amylose did not significantly affect glycaemic response compared to an isoglucidic control matched for starch content. Despite reduced starch digestibility *in vitro*, the *sbeII* bread in this study did not affect glycaemic response when measured remotely and outside of a clinical setting. Additional studies are needed to define target levels of amylose and resistant starch levels to in raw materials (*i.e.* wheat flour) for developing consumer-ready food products that can effectively enhance postprandial glycaemic control.

## Author contributions

Marina Corrado: conceptualization, investigation, methodology, project administration, data curation and formal analysis, writing – original draft preparation, writing – review & editing; George Savva: methodology, data curation, visualisation, formal analysis, writing – original draft preparation, writing – review & editing; Jennifer Ahn-Jarvis: investigation, writing – review & editing; Samson Oyeyinka: investigation, writing – review & editing; Cathrina H. Edwards: conceptualization, methodology, supervision writing – review & editing; Brittany A. Hazard: conceptualization, funding acquisition, methodology, supervision, writing – original draft preparation, writing – review & editing.

## Conflicts of interest

The authors declare no conflicts of interest.

## Abbreviations

CGM: Continuous glucose monitoring
GI: Glycaemic index
QI: Quadram institute
RS: Resistant starch
VAS: Visual analogue scale
WT: Wildtype

## Supplementary Material

FO-016-D5FO03147H-s001

## Data Availability

Annotated code and source data will be made available in the online Zenodo repository, https://doi.org/10.5281/zenodo.17130336. Supplementary information (SI) is available. Supplementary Table 1. Certificate of microbiology analysis. Supplementary Table 2. Proximate analysis of flour. Supplementary Table 3. Nutritional composition and ingredients of Flora Dairy Free Spread. Supplementary Table 4. Questions used for satiety and palatability assessment. Supplementary Table 5. Variance components from the random slope models. Supplementary Fig. 1. Glucose concentration traces during the pre-prandial and postprandial periods. Supplementary Fig. 2. Distributions of sensory outcomes by meal from online questionnaires. See DOI: https://doi.org/10.1039/d5fo03147h.
