# Supplementary material for: Effect of wheat bread with elevated amylose on postprandial glycaemic response: a randomised crossover trial delivered remotely using continuous glucose monitoring
Source: Food Funct. 2025 Nov 24;16(24):9489–503. doi: 10.1039/d5fo03147h (PMC12641596; doi:10.1039/d5fo03147h)

Supplementary Information (SI) for Food & Function.
This journal is © The Royal Society of Chemistry 2025

Supplementary Information

Effect of wheat bread with elevated amylose on postprandial glycaemic response: a randomised crossover trial delivered remotely using continuous glucose monitoring

Supplementary Table 1. *Certificate of microbiology analysis by ALS Laboratories (UK) Limited – Sands Mill, Huddersfield Road, Mirfield, West Yorkshire, WF14 9DQ*

Sample	Method	Test	Result	Unit
sbeII bread	ESGM-M300	Total Viable Count, 2 days	<10	cfu / g
	ESGM-M303	Enterobacteriaceae (presumptive)	<10	cfu / g
	ESGM-M304	Escherichia coli (β-Glucuronidase positive)	<10	cfu / g
	ESGMM307	Coagulase positive Staphylococci (presumptive)	<20	cfu / g
	ESGM-M319	Bacillus cereus (presumptive)	<20	cfu / g
	ESGM-M310	Clostridium perfringens (presumptive)	<10	cfu / g
	ESGM-M308	Yeasts	<20	cfu / g
	ESGM-M308	Moulds	<20	cfu / g
	ESGM-M515	Salmonella sp. (ELISA)	Not detected	/25 g
WT bread	ESGM-M300	Total Viable Count, 2 days	<10	cfu / g
	ESGM-M303	Enterobacteriaceae (presumptive)	<10	cfu / g
	ESGM-M304	Escherichia coli (β-Glucuronidase positive)	<10	cfu / g
	ESGMM307	Coagulase positive Staphylococci (presumptive)	<20	cfu / g
	ESGM-M319	Bacillus cereus (presumptive)	<20	cfu / g
	ESGM-M310	Clostridium perfringens (presumptive)	<10	cfu / g
	ESGM-M308	Yeasts	<20	cfu / g
	ESGM-M308	Moulds	<20	cfu / g
	ESGM-M515	Salmonella sp. (ELISA)	Not detected	/25 g

Supplementary Table 2. *Proximate analysis of flour by ALS Laboratories (UK) Limited – Sands Mill, Huddersfield Road, Mirfield, West Yorkshire, WF14 9DQ*

Parameter	Method	WT control	*sbeII*
Moisture (Loss on Drying) g / 100g	AM/C/801	14.5	13.7
Protein (Nx6.25) g / 100g	AM/C/224	14.9	17.1
Total Fat (NMR) g / 100g	AM/C/1015	0.9	0.6
Ash g / 100g	AM/C/803	0.5	0.7
Total Carbohydrate (by difference) g / 100g	AM/C/901	69.2	67.9
Total Dietary Fibre (based on AOAC method 2017.16) g / 100g	AM/C/309	4.1	5.8
Available Carbohydrate (by difference) g / 100g	AM/C/901	65.1	62.1
Energy kcal / 100g	AM/C/901	336	334
Energy kJ / 100g	AM/C/901	1426	1415
Total Sugar g / 100g	AM/C/1014	0.7	1.2
Starch (by difference) g / 100g	AM/C/901	64.4	60.8
Sodium (ICP-OES) mg / 100g	AM/C/1002	<3	<3
Sodium Expressed as Salt g / 100g	AM/C/1002	<0.01	<0.01
Saturated Fatty Acids g / 100g	AM/C/107	0.19	0.12
Monounsaturated Fatty Acids g / 100g	AM/C/107	0.11	0.08
Polyunsaturated Fatty Acids g / 100g	AM/C/107	0.56	0.37

Supplementary Table 3. *Nutritional composition and ingredients of Flora Dairy Free Spread (obtained using Nutritics database).*

	Per 100g		Per 10 g Serving	
Energy(kcal)	634	kcal	63.4	kcal
Energy(Kj)	2607	kJ	260.7	kJ
Carbohydrate	0.5	g	0.05	g
Protein	0.5	g	0.05	g
Fat	70	g	7	g
Water	28	g	2.8	g
Sugars	0.5	g	0.05	g
Saturated Fat	17	g	1.7	g
Omega3(n-3)	3.9	g	0.39	g
Sodium	360	mg	36	mg
Chloride	545	mg	54.5	mg
Vitamin A	120	ug	12	ug

Ingredients: Plant Oils (Sunflower, Rapeseed, Palm*), Water, Salt, Plant Based Emulsifier (Sunflower Lecithin), Vinegar, Natural Flavouring, Vitamin A, *Flora contributes to the production of sustainable palm oil

Supplementary Table 4. *Questions used for satiety and palatability assessment.*

1. Which study food will you be testing?
2. Are you feeling well today?
3. When was the last time you had something to eat or drink (other than water)?
4. What did you last have to eat and drink?
5. How much could you eat?
6. How strong is your urge to eat?
7. How strong is your thought of food?
8. How would you describe the test meal?
9. Did you finish the entire test meal?
10. If not, approximately how much did you have (eat/drink)?
11. Where there any specific parts of the meal that you left behind?
12. Overall liking:
    a. Aroma (smell)
    b. Flavour (taste)
    c. Sweetness
    d. Bitterness
13. How often would you like to eat this test meal?
14. How was the portion size of the test meal?
15. Do you have any other comments about the test meal?
16. How much could you eat?
17. How strong is your urge to eat?
18. How strong is your thought of food?

Supplementary Table 5. *Variance components from the random slope models*

Measure	Random intercept standard deviation	Random slope standard deviation	Residual standard deviation
**iMax120**	0.634	0.040	0.616
**iMax240240**	0.628	0.060	0.614
**iAUC120**	39.479	27.198	36.676
**iAUC240**	53.638	41.207	59.120

Supplementary Figure 1. *Glucose concentration traces during the pre-prandial and postprandial periods for each participant. The x-axis represents time (minutes) relative to self-reported meal start (black line), and the y-axis shows glucose concentration (mmol/L).*

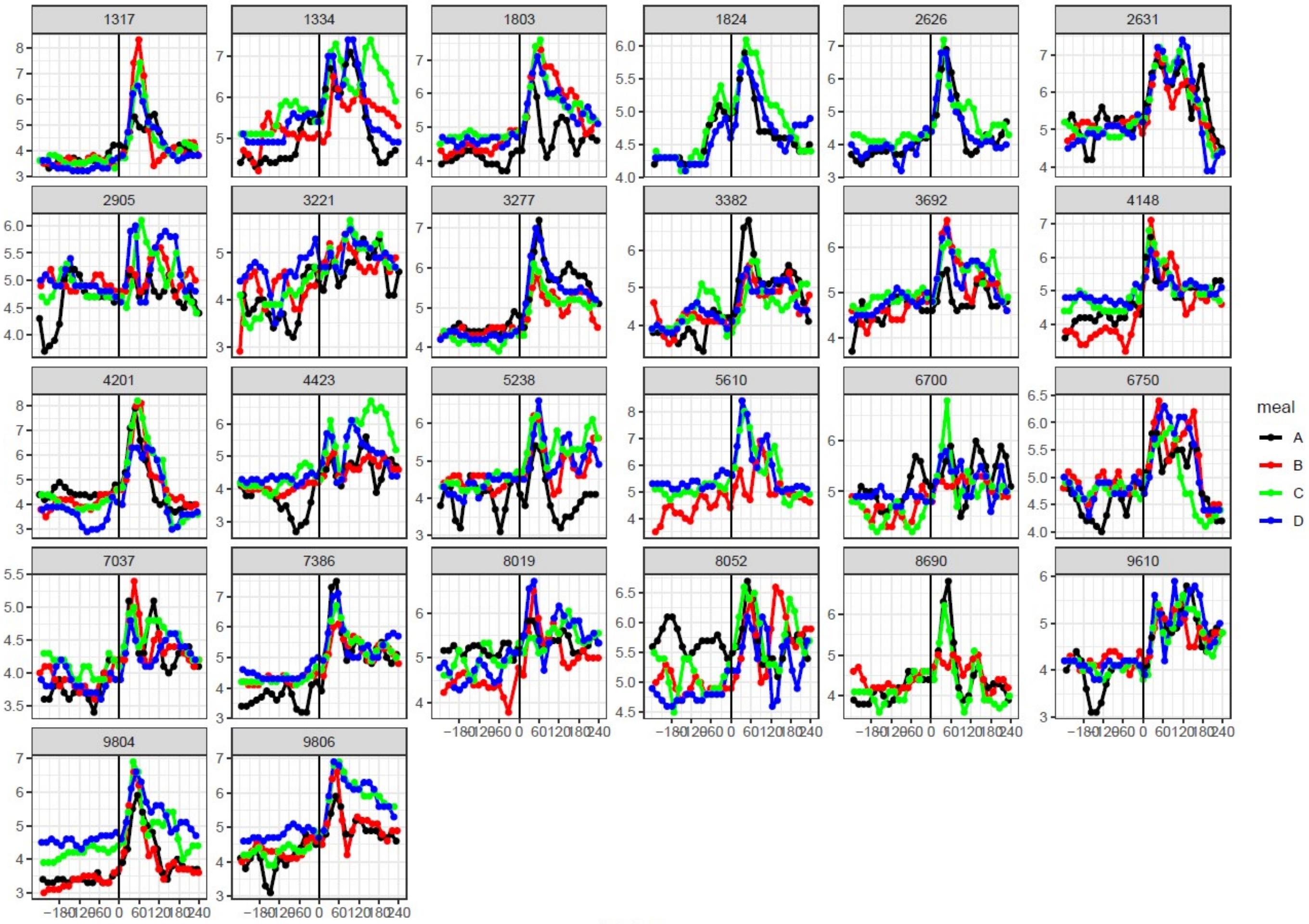

Supplementary Figure 2. *D* [illegible] *ing.* **B.** *'How often would you eat this brea* [illegible]

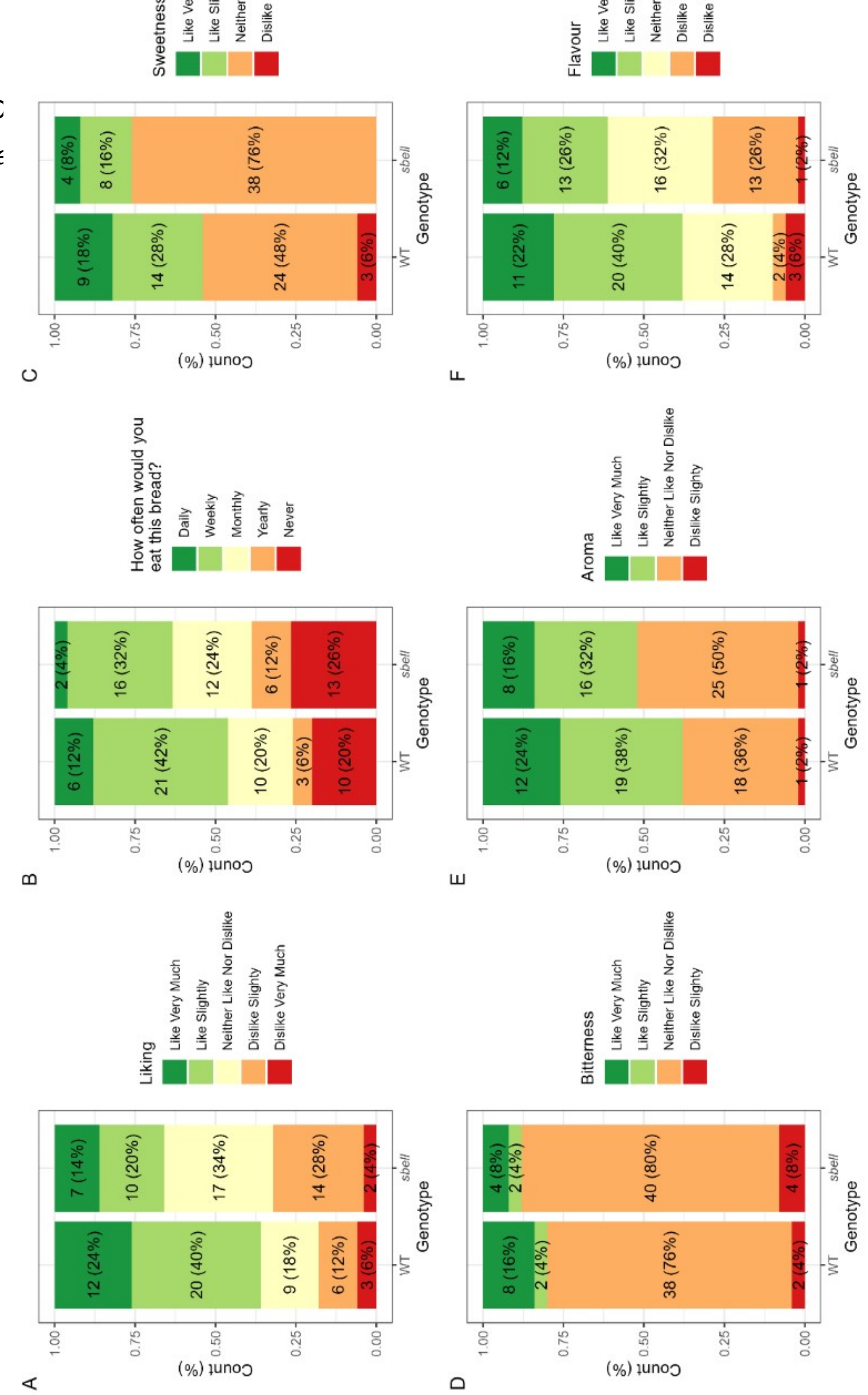

Supplement: FO-016-D5FO03147H-s001 [file FO-016-D5FO03147H-s001.pdf]
